# Assessing Latent Effects of Prenatal Cocaine Exposure on Growth and Risk of Cardiometabolic Disease in Late Adolescence: Design and Methods

**DOI:** 10.1155/2012/467918

**Published:** 2012-12-05

**Authors:** Sarah E. Messiah, Steven E. Lipshultz, Tracie L. Miller, Veronica H. Accornero, Emmalee S. Bandstra

**Affiliations:** ^1^Division of Pediatric Clinical Research, Department of Pediatrics, University of Miami Leonard M. Miller School of Medicine, Miami, FL 33130, USA; ^2^Department of Epidemiology and Public Health, University of Miami Leonard M. Miller School of Medicine, Miami, FL 33130, USA; ^3^Division of Clinical Research, Department of Pediatrics, Batchelor Children's Research Institute, University of Miami Leonard M. Miller School of Medicine, 1580 NW 10th Avenue (D820), Miami, FL 33101, USA; ^4^Division of Neonatology, Department of Pediatrics, University of Miami Leonard M. Miller School of Medicine, Miami, FL 33130, USA; ^5^Division of Pediatric Psychology, Department of Pediatrics, University of Miami Leonard M. Miller School of Medicine, Miami, FL 33130, USA

## Abstract

Prenatal cocaine exposure has been linked to neurocognitive and developmental outcomes throughout childhood. The cardiovascular toxicity of cocaine is also markedly increased in pregnancy, but it is unknown whether this toxicity affects anthropometric growth and the development of cardiometabolic disease risk factors in the offspring across the lifespan. During the early 1990s, the Miami Prenatal Cocaine Study enrolled a cohort of 476 African American children (253 cocaine-exposed, 223 non-cocaine-exposed) and their biological mothers at delivery in a prospective, longitudinal study. The MPCS has collected 12 prior waves of multidomain data on over 400 infants and their mothers/alternate caregivers through mid-adolescence and is now embarking on an additional wave of data collection at ages 18-19 years. We describe here the analytical methods for examining the relationship between prenatal cocaine exposure, anthropometric growth, and cardiometabolic disease risk factors in late adolescence in this minority, urban cohort. Findings from this investigation should inform both the fields of substance use and cardiovascular research about subsequent risks of cocaine ingestion during pregnancy in offspring.

## 1. Introduction

 Prenatal cocaine exposure (PCE) has been linked to numerous adverse effects throughout childhood, including cognitive, language, neurodevelopmental, and behavioral deficits [1–5]. Although the implications of PCE on neurological and behavioral health have been rather extensively studied, the implications of PCE on physical health, especially in late adolescence, have not been emphasized. 

The cardiovascular toxicity of cocaine is markedly increased in both mother and child during pregnancy [6, 7]. Maternal complications of cocaine use during pregnancy include premature labor, placental abruption, uterine rupture, cardiac dysrhythmias, hepatic rupture, cerebral ischemia or infarction, and death [6–9]. Cardiac manifestations in children exposed to cocaine in utero include arrhythmias and malformations, such as atrial and ventricular septal defects, hypoplastic right or left heart, absent ventricle [10], coarctation of the aorta, aortic valve prolapse, patent ductus arteriosus, and peripheral pulmonary stenosis [11]. In some children, these cardiovascular abnormalities are associated with congestive heart failure, cardiorespiratory arrest, and death [10–14].

Given these cocaine-related cardiovascular complications in infancy, it is possible that PCE also affects growth and the development of cardiometabolic disease risk factors, such as elevated blood pressure, lipids, C-reactive protein (CRP), and insulin insensitivity, later in life. Heart disease and diabetes are both major causes of mortality (ranked first and sixth, resp.) in the United States, killing more than 700,000 people each year [15]. Almost 5% of women report using an illicit drug, including cocaine, during pregnancy (and almost 11% of nonpregnant women in the past month) [16], thereby exposing tens of thousands of fetuses to cocaine each year. If PCE does, in fact, have cardiovascular implications later in life, the personal and public health consequences could be serious.

 Thus, there is a compelling need for assessing the potential impact of in utero cocaine exposure on cardiometabolic health over the lifespan. This paper describes the study design and methods for such a longitudinal study using data from the prospective and ongoing Miami Prenatal Cocaine Study (MPCS) cohort. Assessments from birth to mid-adolescence were funded by the National Institute on Drug Abuse (NIDA; R01 DA 006556). The 16/17-year assessments and ongoing 18/19-year assessments of this cohort have been supported by a clinical project, “Sex and Gender Influences on Drug Involvement in Adolescence,” conducted within the University of Miami's Specialized Center of Research (SCOR) on Addiction and Health in Women, Children, and Adolescents, which is co-funded by the Office of Research on Women's Health (ORWH) and NIDA (P50 DA 024584). 

In the current study, the MPCS and SCOR data are being augmented with additional measures and analyses to determine whether PCE is associated with long-term perturbations in anthropometric growth and the development of risk factors for cardiometabolic disease in late adolescence. The design and methods for this investigation, which is supported by a NIDA Mentored Career Development Award to the first author (K01 DA 026993), is the subject of this report. The results will be published separately. 

## 2. Research Design and Methods

Children and adolescents enrolled in the Miami Prenatal Cocaine Study comprise one of the oldest, largest, and well-characterized, single-site cohorts of this type in the nation. During 33 months in the early 1990s, 476 African American infants (253 cocaine-exposed and 223 non-cocaine-exposed) were enrolled in the prospective study at delivery (Table 1). At enrollment, the biological mothers participated in a detailed, confidential postpartum interview about their drug use and gave permission for collection of maternal and infant urine and meconium for assays of cocaine and other drugs (Table 2). This longitudinal study has been approved annually by the Institutional Review Board and is conducted under a federal Department of Health and Human Services Certificate of Confidentiality. 

The MPCS sample is relatively homogeneous with respect to important demographic factors (e.g., African American race-ethnicity, full-term gestational age, urban delivery setting, and low socioeconomic status). Comprehensive and standardized assessments of developmental, neuropsychological, educational, behavioral, and social-environmental domains at 12 time points between birth and age 16 or 17 years permit making longitudinal interpretations. 

 The main MPCS protocol has been focused on the suspected neurodevelopmental outcomes (Table 3), and the related SCOR clinical project, which includes follow-up visits at ages 16 or 17 years and at ages 18 or 19 years, primarily examines sex differences in adolescent drug involvement in the original MPCS cohort (Tables 4 and 5). The current subprotocol (K01 DA 026993) is focusing on assessing anthropometric growth and cardiometabolic disease risk factors. Specifically, we will compare prenatally cocaine-exposed and nonexposed 18- to 19-year-olds on anthropometric measures (height, weight, body mass index, waist circumference, and body composition) and cardiometabolic risk factors (fasting glucose, insulin, lipids, highly sensitive (hs)CRP, and blood pressure). We will then determine the specific effects of both prenatal and postnatal cocaine exposures in a repeated-measures multivariate analysis over the entire period, controlling for effect modifiers (e.g., prenatal exposure to alcohol, tobacco, marijuana, stress, anxiety, and depression in the adolescents and their mothers or alternate primary caregivers as measured by the batteries included in Tables 3–5) on overweight (≥85th percentile for body mass index (BMI) for age and sex) and obesity (≥95th percentile for BMI for age and sex), underweight, and the presence of risk factors for metabolic syndrome, a condition defined as the presence of three or more of the cardiometabolic risk factors listed.

 Below, we summarize the MPCS, which forms the centerpiece for this project, and describe the subprotocol methodology for adding cardiometabolic disease risk factor assessments of 18- to 19-year-olds. 

### 2.1. The Miami Prenatal Cocaine Study

The MPCS is an ongoing, NIDA-funded investigation of the effects of maternal gestational use of cocaine and other drugs on multidomain outcomes of the offspring from birth through adolescence. The original cohort consisted of 476 full-term infants enrolled prospectively at birth (253 cocaine-exposed and 223 non-cocaine-exposed, of whom 147 were drug-free and 76 were exposed to varying combinations and amounts of alcohol, tobacco, and marijuana). Biological mothers of these follow-up participants were negative for HIV infection at enrollment and had no evidence of opioid, amphetamine, barbiturate, benzodiazepine, or phencyclidine use during pregnancy. 

The cohort was drawn from a survey of 1,505 African American women delivering full-term infants at the University of Miami Jackson Memorial Hospital between 1990 and 1993. Prenatal cocaine exposure was determined by maternal self-report (Table 2) or positive cocaine or cocaine metabolite assay in maternal or infant urine or meconium (see below). The sample was intentionally restricted to mothers of low socioeconomic status, inner-city residence, and African American race to improve statistical power and covariate control. None of the infants had major congenital malformations or disseminated congenital infection. 

### 2.2. Measures of Prenatal Drug Exposure at Birth

#### 2.2.1. Maternal Self-Report

A structured postpartum interview to ascertain maternal drug use was conducted by separate research staff distinct from the infant and child assessment examiners. To enhance timeline recall, periods were outlined and anchored to important calendar dates. Drug use during each trimester of pregnancy was assessed with a standardized, structured interview. Questions for each trimester included the number of weeks the drug was used, the usual number of days per week, and the usual dose per day for each drug of interest. Dosage was recorded in number of cigarettes smoked per day, number of marijuana joints smoked per day, and number of standard drinks for each type of alcohol (i.e., beer 12 oz., wine 5 oz., and liquor 1.5 oz.) as defined by Schneiderman [91]. Cocaine dosage was recorded as the number of rocks of crack cocaine or lines of powder cocaine used per day. 

Total drug exposure was calculated for each drug in the prepregnancy, trimester-specific, and total pregnancy periods by multiplying the usual dosage per day by the usual days per week by the number of weeks used in each period of interest. In the analyses for the current study of growth and cardiometabolic risk, total pregnancy exposure to alcohol, tobacco, and marijuana will be used as covariates. Self-reported cocaine use and toxicology data were used to determine group assignment (Table 2). In addition, a latent construct, also based on self-report and toxicology data, has been used to indicate the level or severity of cocaine exposure for analytic purposes [1].

#### 2.2.2. Biological Markers of Cocaine Use

Urine and meconium samples were initially screened with EMIT for the cocaine metabolite, benzoylecgonine (BE), at a cut-off of 150 ng/mL in urine and 150 ng/g in meconium. Positive assays were confirmed by gas chromatography/mass spectrometry [92]. Urine specimens were screened with EMIT for marijuana (cannabinoids), opiates, amphetamines, barbiturates, benzodiazepines, and phencyclidine. Meconium specimens were also assayed by EMIT for marijuana and opiates. Among follow-up participants, 100% had at least one of the three delivery biological specimens, 96% had at least two, and 68% had all three. 

### 2.3. Neurodevelopmental Measures 

Infant neurobehavior and development were assessed with the Brazelton Neonatal Behavioral Assessment Scale [93] at birth and 1 month of age and with the Bayley Scales of Infant Development [94] at 4, 8, 12, 18, and 24 months of age. Cranial ultrasound was performed at birth and at 1 and 4 months of age. Preschool children between 3 and 5 years of age were assessed via standardized measures for cognition; language development; emerging self-regulation of attentional, behavioral, and emotional processes; motor skills; and the quality of the caregiving environment [95–98]. At ages 7 and 12 years, participants' neuropsychological functioning (attention, executive functioning, memory), language skills, and behavior regulation were assessed. The quality of the environment was assessed by collecting data on caregiver substance use and psychological functioning, parenting qualities, family functioning, exposure to violence, and neighborhood economic conditions (Table 3) [17–64]. As part of the SCOR clinical project, the MPCS cohort was also assessed at ages 16 or 17 (Table 4) [65–86] and (currently) at 18 or 19 years (Table 5) [62, 70, 71, 80, 83, 87–90].

Assessments used in the 16/17- and 18/19-year visits measure major domains of drug involvement, stress and coping, risk-taking propensity, and reward decision making, along with several others (see Tables 4 and 5). Details of selected standardized measures administered at one or both of the adolescent visits are as follows.

**Drug Involvement**

The Communities That Care Youth Survey [62] assesses youth report of alcohol, tobacco, and other drug use and evaluates risk and protective factors often related to substance use within the domains of family, peers, school, and community.

**Stress and Coping**

The Trier Social Stress Test-Children (TSST-C) [65] assesses physiological reactivity to an acute stressor. This task involves 5 minutes each of preparation, public speaking, and a mental arithmetic task. Salivary-free cortisol levels are assessed at 5 intervals: prior to the task, immediately following the task, and at 3 10-minute intervals thereafter. The Life Events Questionnaire-Adolescence (LEQA) [66] is a 67-item questionnaire composed of statements briefly describing life events, which the respondent rates as occurring or not in the past year (yes/no). Events are categorized with regard to (a) discreteness of onset: discrete, chronic, or ambiguous; (b) desirability: positive, negative, or ambiguous; and (c) independence: out of the child's control, child had some control, or control depends on the context of the event. Additionally, the Childhood Trauma Questionnaire (CTQ) [84] assesses retrospectively the occurrence of abuse and neglect in childhood and adolescence. This questionnaire includes 28 items, rated on a 5-point Likert scale, that address 5 types of maltreatment: physical abuse, sexual abuse, emotional abuse, physical neglect, and emotional neglect. Finally, to assess more chronic daily hassles, the 32-item Urban Hassles Index (UHI) [67] was developed and has been tested specifically with ethnic minority youth. Maladaptive coping in response to stress may also influence drug use. To this end, the Adolescent Coping Orientation for Problem Experiences (ACOPE) [68] assesses coping patterns along 12 subscales (ventilating feelings, seeking diversions, developing self-reliance and optimism, developing social support, solving family problems, avoiding problems, seeking spiritual support, investing in close friends, seeking professional support, engaging in demanding activity, being humorous, and relaxing).

**Internalizing and Externalizing Behavior**

To assess diagnostic criteria for DSM-IV disorders in youth, the DISC Predictive Scales (DPS) [49] was administered to the caregiver and adolescent. The DPS was selected due to its brevity and ability to detect and discriminate disorders in adolescents. The dimensional nature of emotional and behavioral problems is also being assessed via computerized versions of the well-known Achenbach Youth Self-Report (YSR) [69] and Child Behavior Checklist (CBCL) [69], administered to the adolescent and caregiver, respectively. These questionnaires, which measure social competence and internalizing and externalizing symptoms in children and youth, have extensive normative data and excellent reliability and validity. Anxiety and depression are often associated with substance use. As such, the Beck Depression Inventory-II (BDI-II) [70] and Beck Anxiety Inventory [71], self-report questionnaires of the presence and severity of depressive and anxiety symptoms according to DSM-IV criteria in adults and adolescents, are administered.

**Risk-Taking Propensity**

The Balloon Analogue Risk Task (BART) [73] is a computerized, laboratory-based measure simulating risky behavior. In the adolescent version, the participant pumps up a balloon figure presented on the computer screen, earning one point for each pump. He/she can bank the points earned for that particular balloon at any time and move on to the next balloon. However, if the balloon explodes before the points are banked, earnings for that particular balloon are lost. Balloons (30 in all) pop at varying, unpredictable points. The score for the BART is the average number of pumps across balloons, excluding those balloons that explode; the number of pops will also be examined. The BART has been correlated with self-reported engagement in risky behavior (e.g., drug use, gambling, fighting, and risky sexual behavior) by adolescents. The Wall Task [74], a very brief cartoon-based measure of risk-taking has been shown to predict initiation of cocaine use (and other drugs) in early adulthood. Delay discounting [75–77] or temporal discounting refers to tendency to overvalue immediate rewards compared to delayed rewards (or to “discount” the value of delayed rewards) and has been interpreted as a form of impulsivity. In this task, participants are asked to choose between an amount of money (hypothetical) delivered now or an amount of money (hypothetical) delivered after a certain amount of time. Varying amounts of money and delay intervals are presented over multiple trials. These behavioral tasks are supplemented with two self-report measures of sensation-seeking and impulsivity: the Impulsive Sensation Seeking Scale (from Zuckerman-Kuhlman Personality Questionnaire, ZKPQ) [64] and the Eysenck Impulsivity Scale [78].

**Reward Decision Making**

The Wheel of Fortune [79] was developed to examine the behavioral correlates of motivated behavior and reward processes. It involves a decision-making challenge by having participants choose during trials between a low probability of a large reward and a high probability of a small reward. The WOF probes the separate steps of reward-related decision-making, allowing for the assessment of (1) patterns of choice selection in conditions with varying levels of risk, (2) confidence in favorable outcomes, and (3) responses to feedback. This is important given that decision-making deficits in general have been demonstrated in adolescents at risk for substance abuse.

### 2.4. Data Collection at the 18/19-Year Study Visit

#### 2.4.1. Attention and Executive Functioning

The Cambridge Neuropsychological Testing Automated Battery (CANTAB) [90] is a comprehensive, computerized assessment of neuropsychological functioning. Though originally developed for use with the elderly, the CANTAB has since been utilized with children and adults of ages 4 through 90. The advantages of the CANTAB include standardized computer administration, use of nonverbal stimuli, engaging, game-like quality, and tests graded in difficulty to capture a wide range of abilities. In the current study, 14 tests from the Executive Function, Attention, and Decision Making and Response Control domains of the CANTAB are administered. The Executive Function battery (Intra-Extra Dimensional Set Shift; One Touch Stockings of Cambridge; Stockings of Cambridge; Spatial Span; Spatial Working Memory) measures abilities such as attentional set shifting, rule acquisition, spatial planning, working memory, motor control, and strategy use. The Attention tests (Choice Reaction Time, Match to Sample Visual Search, Reaction Time, Rapid Visual Information Processing, and Simple Reaction Time) measure different aspects of attention and reaction time. The Decision Making battery includes four tests (Affective Go/No-go; Cambridge Gambling Task; Information Sampling Task; Stop Signal Task), tapping information processing biases for positive and negative stimuli, impulse control and risk taking in decision making, and ability to inhibit a prepotent response.

#### 2.4.2. Anthropometric Measures

 Trained nurses or research assistants blinded to cocaine exposure status perform standard anthropometric measures of stature (recumbent length or standing height, depending on age), weight, and head circumference at birth, at each developmental assessment visit (at ages 1, 4, 8, 12, 18, and 24 months and at 3, 5, 7, 12, and 16 or 17 years), and at interim visits. Anthropometric and clinical measures in the protocol for 18- and 19-year-olds include standing height, measured with a Detecto Physician's scale (Cardinal Scale Manufacturing Co., Webb City, MO, USA) with height rod with a vertical backboard and a movable headboard; weight, measured in pounds and converted to kilograms (nearest 1/10); waist circumference, measured to the nearest 0.1 cm at the navel at the end of gentle exhalation; hip circumference, measured to the nearest 0.1 cm at the maximum circumference over the buttocks; mid-arm circumference, measured to the nearest 0.1 cm. Waist and hip circumferences are measured using a nonstretchable plastic tape measure according to standard methods [99].

#### 2.4.3. Skinfold Measurements

 Subscapular and suprailiac skinfold thicknesses are measured to the nearest millimeter (mm) to assess truncal subcutaneous adiposity [99]. The protocol for 18- and 19-year-olds also includes skinfold measurements of the triceps and biceps, measured to the nearest mm. 

 All anthropometric measures are taken three times and the average of the three measures is used for data analysis. Height and weight are converted to sex-specific BMI-for-age percentile values according to the 2000 CDC growth charts [99].

#### 2.4.4. Cardiometabolic Measures

Within the MPCS, the subprotocol for 18- and 19-year-olds includes testing for several cardiometabolic biomarkers, using blood drawn by venipuncture after a 12-hour fast. All blood chemistry assays are performed by an automated analyzer (Roche Cobas-Mira, Indianapolis, IN, USA), using commercially available kits according to the manufacturer's instructions. Instrument setup, run procedures, and maintenance policies are strictly applied according to the manufacturer's instructions.

**Fasting Insulin and Glucose**

Blood glycohemoglobin, fasting plasma glucose, and serum insulin are measured after an overnight fast (blood is drawn when participants arrive for their visit, before the assessments begin) [100]. A fasting plasma glucose greater than 100 mg/dL is considered abnormal as defined by the American Diabetes Association [101]. Glucose and insulin values will be used in a homeostasis model assessment of insulin resistance (HOMA [IR]) to calculate an insulin resistance score for each participant [101]. HOMA (IR) is a surrogate index widely used to study the role of insulin sensitivity or resistance in associated disease states and is defined as fasting plasma insulin (*μ*IU/mL) × fasting plasma glucose (mmol/L)/22.5.

**Triglycerides**

High serum levels of triglycerides have been linked to atherosclerosis and are a component of metabolic syndrome [102]. Abnormal values will be reported as sex- and age-specific national percentile estimates [102].

**Total Cholesterol**

Because they signal hyperlipidemia, blood lipid levels are an important indicator of cardiovascular disease risk [102]. As with triglycerides, abnormal values will be reported as sex- and age-specific national percentile estimates [102].

**High- and Low-Density Lipoprotein (HDL and LDL) Cholesterol**

High levels of HDL seem to protect against cardiovascular disease, and low levels are associated with an increased risk of heart disease [102]. As with triglycerides, abnormal total, HDL, and LDL cholesterol values will be reported by sex- and age-specific national percentile estimates [102].

**Highly Sensitive C-Reactive Protein (hs)CRP**

C-reactive protein is considered one of the best measures of inflammation, which is one of the body's responses to chronic conditions, such as arthritis, and to environmental exposures to agents, such as tobacco smoke. The American Heart Association and the US Centers for Disease Control and Prevention have stated that in population studies of risk of cardiovascular disease in adults, an (hs)CRP concentration greater than 3.0 mg/L is considered high risk [103]. As the current assessment of the MPCS cohort is at age 18-19 years, the adult cut-off for (hs)CRP will be utilized.

#### 2.4.5. Definition of Metabolic Syndrome

 Participants who have 3 or more of the 5 following conditions will be classified as having the syndrome: a fasting blood glucose concentration greater than 100 mg/dL [101], a waist circumference greater than the 90th percentile adjusted for age, sex, and race [104], a systolic or diastolic blood pressure greater than the 90th percentile for age and sex [105], hypertriglyceridemia defined as greater than the 90th percentile for age, race, and sex [102], and low HDL cholesterol defined as less than the 5th percentile for age, sex, and race [102].

#### 2.4.6. Lifestyle Risk Factors

 Via a written questionnaire, adolescent participants reported their daily eating habits and physical activity level. These items were adapted from the Centers for Disease Control and Prevention's Youth Risk Behavior Surveillance (YRBS) Questionnaire [106]. The YRBS monitors of health-risk behaviors priority and the prevalence of obesity and asthma among youth and young adults by implementing a national school-based survey and state, territorial, tribal, and district surveys. Specifically, the lifestyle risk factor questions focus on how often certain foods (fruits, vegetables, fast food, etc.) were consumed in a 7-day period and how many minutes per day and per week the adolescent participated in strenuous (leading to heavy breathing or sweating) physical activity.

### 2.5. Analytic Plan

To achieve the specific and primary aims of the subprotocol described here, longitudinal analyses will focus on differences in latent growth trajectories between prenatally cocaine-exposed and non-cocaine-exposed participants. In a cross-section analysis, the influence of prenatal and postnatal exposure to drugs (alcohol, tobacco, and other drugs) and the development of cardiometabolic disease risk factors will be estimated. Metabolic syndrome variables added to the protocol for 18- and 19-year-olds will be log-transformed to achieve a more normal distribution before analyses (all females who are pregnant or who have had babies in the last 6 months will be excluded). Growth curves for metabolic syndrome variables versus age (using both linear and nonlinear terms) will be determined for each participant in a random-effects model. Pairwise correlations of any two variables (prenatal cocaine exposure, cardiometabolic risk variables) will be examined using the appropriate correlation analysis. The differences in the Pearson correlation coefficients between sexes and between follow-up years will be tested by Fisher's *z* transformation. 

Additionally, secondary analysis will include examining the (potentially bidirectional) relationship between primary outcomes (anthropometric growth, cardiometabolic disease risk factors) and several neurodevelopmental measures. For example, the relationship between depression and anxiety (measured by the Beck Depression Inventory-II [BDI] and Beck Anxiety Inventory (BAI)) and all growth and cardiometabolic measures will be examined. 

## 3. Discussion 

We describe here the research and analytical methods for examining the relationship between prenatal cocaine exposure, anthropometric growth, and cardiometabolic disease risk factors in late adolescence in this minority, urban cohort. By examining child and adolescent growth and development of cardiometabolic disease among those exposed to cocaine in utero, it is expected that prenatally exposed offspring may be shown to develop abnormal growth trajectories, particularly catch-up growth, which may result in increased risk for associated cardiometabolic disease risk factors. Findings from this investigation should inform both the fields of substance use and cardiovascular research about subsequent risks of cocaine ingestion during pregnancy in offspring. 

Elsewhere, our group has reviewed the literature that supports the hypothesis that prenatal cocaine exposure impacts latency health outcomes via various biological mechanisms, and the cardiovascular system in particular [107]. For example, vascular and hemodynamic functions are partially programmed in early life and are thus vulnerable to prenatal exposures, with potential impact to adverse vascular aging and arterial stiffening in later life. Additionally, prenatal cocaine exposure has resulted in fetal cardiovascular alterations including diastolic function, heart rate variability, and transient myocardial ischemia; but how this affects health, and other body systems (e.g., endocrine and renal, etc.) in later life is largely unknown [107]. This determination is particularly important among African Americans adolescents who are already at high risk for type 2 diabetes and cardiovascular disease [108]. Strategies for preventing and treating these effects should be developed to reduce the overall burden of disease for individuals and the accompanying costs to the health care system.

### 3.1. Potential Limitations

Several methodological limitations are intrinsic to the study of prenatal drug exposures, including the nonexperimental design and the difficulty of separating the influences of such exposures from those of a myriad of other factors. Nonetheless, the potential teratologic effects of these drugs are of interest, and efforts are made to statistically control for potential confounding variables to attempt to isolate in utero exposure to cocaine and other drugs as contributing factors within the context of the multiple risks which may be involved in shaping the cardiometabolic outcomes of drug-exposed children. 

The MPCS sample was restricted to full-term, healthy African American infants living in generally low-income areas of the city. Although this restricted sampling was done to improve statistical control, such limitations may pose certain challenges in interpreting the findings. For example, excluding infants born prematurely and with serious medical difficulties may eliminate the more heavily cocaine-exposed children from the sample. Also, findings from the MPCS may not generalize to other populations, such as those from different racial or ethnic backgrounds and socioeconomic status.

The research examines the influence of PCE on the risk of cardiometabolic disease risk in late adolescence. However, this relationship may not encompass all the possible risk and protective factors relevant to the long-term effects of prenatal drug exposure. For example, the genes or functional polymorphisms that affect the development of cardiovascular disease and type 2 diabetes need to be explored. Thus, the apparent effects of prenatal drug exposure may actually be caused by an underlying, if yet undiscovered, genetic susceptibility.

## 4. Conclusions

The results of this study, using longitudinal and cross-sectional data from the MPCS to assess growth patterns, obesity, overweight, and hypertension, as well as late adolescent cardiometabolic blood tests, should help to determine whether in utero exposure to cocaine has short- and long-term effects on physical development, especially on the cardiovascular and endocrine systems. Long-term follow-up of the participants in the MPCS as well as other representative cohorts will allow the determination of whether any adverse physical and cardiometabolic effects appear only after long latency periods, much like the cardiovascular complications in survivors of childhood cancers treated with anthracyclines which may not appear for decades. 

## Figures and Tables

**Table 1:** Characteristics of infants with and without prenatal cocaine exposure and their mothers at enrollment in the Miami Prenatal Cocaine Study.

Characteristic	Non-cocaine-exposed infants	Cocaine-exposed infants
	(*n* = 223)	(*n* = 253)
Maternal characteristics		
Maternal age, mean (SD), y*	23.8 (5.4)	28.7 (4.8)
Education, mean (SD), y	11.3 (1.4)	11.2 (1.5)
Unemployed, %*	82	95
Never married, %	89	90
Primigravida, %*	23	6
Prenatal care ≥ 4 visits, %*	83	68
Infant characteristics		
Birth weight, mean (SD), g*	3303 (504)	2971 (474)
Birth length, mean (SD), cm*	50.7 (2.3)	48.9 (2.5)
Birth head circumference, mean (SD), cm*	33.8 (1.5)	33.0 (1.6)
Gestational age, mean (SD), weeks*	39.7 (1.4)	39.4 (1.4)
Male, %	50	48

*P < 0.01.

**Table 2:** Self-reported drug use during pregnancy among women giving birth to infants with or without prenatal cocaine exposure in the Miami Prenatal Cocaine Study.

Drug	Mothers of non-cocaine-exposed infants (*n* = 223)		Mothers of cocaine-exposed infants (*n* = 253)	
	Total drug exposure, median (min to max)*	% Using (*n*)	Total drug exposure, median (min to max)*	% Using (*n*)
Alcohol (number of drinks)^†^	54 (2 to 1680)	30.9 (69)	96 (1 to 5226)	66.8 (169)
Tobacco (number of cigarettes)^†‡^	854 (1 to 5880)	17.0 (38)	2184 (1 to 8820)	73.5 (186)
Marijuana (number of joints)^†^	28 (1 to 807)	11.7 (26)	24 (1 to 1320)	45.1 (114)
Cocaine/crack (number of lines/rocks)	*⋯*	*⋯*	134 (1 to 19600)	68.4 (173)

*Median values based only on mothers reporting use, calculated using total exposure composites: (number of weeks used) × (usual number of days per week) × (usual dose per day).

^†^ *P* < 0.01, between-group comparisons of percentage of maternal drug use (columns 2 and 4).

^‡^ *P* < 0.05, between-group comparisons of median maternal drug use (columns 1 and 3).

**Table 3:** Miami Prenatal Cocaine Study multidomain assessments from preschool to early adolescence.

3-year exam	5-year exam	7-year exam	12-year exam (2 days)
Development, cognition, and achievement			

McCarthy Scales of Children's Abilities [17]	Wechsler Preschool and Primary Scales of Intelligence-Revised (WPPSI-R) [18] Peabody Developmental Motor Scales [19] Developmental Test of Visual Motor Integration [20]	Wechsler Intelligence Scale for Children-3rd Edition (WISC-III) [21] Wechsler Individual Achievement Test (WIAT) [22]	WISC-III [21] WIAT-II [23] Guide to Assessment of Test Session Behavior (GATSB) [24]

Language			

CELF-P [25] Peabody Picture Vocabulary Test-R [26] Behavioral Audiology	CELF-P [25] Woodcock-Johnson Test of Cognitive Ability—Auditory Processing [27]		CELF-3 [28] Comprehensive Test of Phonological Processing [29] SCAN-A [30]

Neuropsychological functioning			

		NEPSY: a developmental neuropsychological assessment (domains: attention/executive function, language, sensorimotor, visuospatial, and memory) [31]	NEPSY (tower, visual and auditory attention, visuomotor) [31] Wisconsin Card Sort [32] Stroop Color Word Test [33] Trail Making Tests, A and B [34] Rey-Osterrieth [35] WRAML (memory) [36]; verbal fluency (FAS) [37] Finger tapping [38]; Grooved pegboard [39]

Behavioral health			

*Child measures* Exploratory Box [40] Mastery Motivation [41] Tester Behavior Ratings	*Child measures* Test of Variables of Attention (TOVA) [42] Delay Task [43] Tester Behavior Ratings	*Child measures* Conners CPT (attention) [44] Tester Behavior Ratings	*Child measures* IVA CPT (attention) [45] Tester Behavior Ratings Achenbach Youth Self-Report (YSR) [46] Children's Depression Inventory (CDI) [47] Revised Children's Manifest Anxiety Scale (RCMAS) [48] DISC Predictive Scales (DPS) [49] ADHD Tester Behavior Observation
*Caregiver report* Achenbach CBCL 2/3 [50] Conners ADHD Rating [51]	*Caregiver report* Achenbach CBCL 4/18-Parent [52] Computerized Diagnostic Interview Schedule for Children (C-DISC) [53] Scales of Independent Behavior-Revised (SIB-R) [54]	*Caregiver report* Diagnostic Inventory for Children and Adolescents (DICA) v.4 [55] Achenbach CBCL-Parent [52]	*Caregiver report* DICA-4 [55] Achenbach CBCL-Parent [52] Adaptive Behavior Assessment Scale (ABAS) [56] Behavior Rating Inventory of Executive Function (BRIEF) [57] DISC Predictive Scales (DPS) [49]
			*Teacher report* Achenbach Teacher Form (TRF) [46] Social Skills Rating System [58] BRIEF [57]
			Communities That Care Youth Survey (Drug Use/Risks) [62] Adolescent Sexual Activity Index [64]

Parent child interaction			

Free play & toy pick-up [59] Home Observation for Measurement of the Environment (H.O.M.E.) [60]	The Teaching Tasks [59]		

Child risk factors			

		Things I Have Seen and Heard: A Violence Exposure Interview [61]	Things I Have Seen and Heard [61] “What's Happening?” Interview [63] Sensation Seeking Scale [64]

**Table 4:** Behavioral health assessments of the adolescents and caregivers at the 16/17-year visit.

Domain	Behavioral health measures	Subject report or source	Caregiver report or source
Drug involvement Risk and protective factors	Communities That Care Youth Survey (CTC) with supplemental drug involvement questions [62]	×	
	Toxicology assays (hair and urine)	×	

Stress and coping	Trier Social Stress Test-Children (TSST-C) [65]	×	
	Life Events Questionnaire-Adolescence (LEQ-A) [66]	×	
	Urban Hassles Index (UHI) [67]	×	
	Adolescent Coping Orientation for Problem Experiences (ACOPE) [68]	×	

Psychological status emotional, behavioral, and adaptive functioning	DISC Predictive Scales (DPS) DSM-IV Diagnoses*-*Adolescent measure [49]	×	×
	Achenbach Child Behavior Checklist 4–18 (CBCL) [69]		×
	Achenbach Youth Self-Report (YSR) [69]	×	
	Beck Depression Inventory-II (BDI) [70]	×	
	Beck Anxiety Inventory (BAI) [71]	×	

Self-esteem	Harter Self-Perception Profile for Adolescents (SPPA) [72]	×	

Risk-taking propensity	Balloon Analogue Risk Task (BART) [73]	×	
	Wall Task [74]	×	
	Delay Discounting [75–77]	×	
	Sensation Seeking Scale (from Zuckerman-Kuhlman Personality Questionnaire, ZKPQ) [64]	×	
	Eysenck Impulsivity Scale [78]	×	

Reward decision making	Wheel of Fortune [79]	×	

Risky sexual behavior	Adolescent Sexual Activity Index (ASAI, modified) [80]	×	

Health and development	Physical exam, growth, and self-report pubertal staging [81, 82]	×	
	Perinatal CARE Program Adolescent Health and Service Utilization Survey	×	×

Psychosocial/demographic history	Perinatal CARE Program Psychosocial-Adolescent Version	×	
	Perinatal CARE Program Psychosocial Interview-Parent Version		×

Caregiver drug use	Addiction Severity Index-5th Edition: Alcohol/Drug Section (modified) [83]		×
	Toxicology assays (hair and urine)		×

Caregiver psychological status	Beck Anxiety Scale (BAI) [71]		×
	Beck Depression Inventory-II (BDI) [70]		×
	Childhood Trauma Questionnaire (CTQ) [84]		×

Family functioning	Family Environment Scale (FES) [85]		×

Parental monitoring and neighborhood characteristics	“What's Happening?” Interview[63]		×

Violence exposure	NIMH Survey of Exposure to Community Violence [86]		×

**Table 5:** Behavioral health assessments of the adolescents and caregivers at the 18/19-year visit.

Domain	Measures	Subject report or source	Caregiver report or source
Drug involvement Risk and protective factors	Communities That Care Youth Survey (CTC) with supplemental drug involvement questions [62]	×	
	Toxicology assays (hair and urine)	×	

Psychological status Emotional, behavioral, and adaptive functioning	Achenbach Adult Self-Report (ASR) [87]	×	
	Achenbach Behavior Checklist (ABCL) [87]	×	×
	Beck Depression Inventory-II (BDI) [70]	×	
	Beck Anxiety Inventory (BAI) [71]	×	
	The Multi-Dimensional Scale of Perceived Social Support [88, 89]	×	
	Childhood Trauma Questionnaire (CTQ) [84]	×	

Attention, executive functioning and decision making	Cambridge Neuropsychological Testing Automated Battery (CANTAB) [90]	×	

Risky sexual behavior	Adolescent Sexual Activity Index (ASAI, modified with supplemental questions on risky sexual behavior) [80]	×	

Physical/sexual health and development	Physical exam, growth	×	
	Perinatal CARE Program Adolescent Health and Service Utilization	×	×
	HIV/STD Testing	×	

Psychosocial/demographic history	Perinatal CARE Program Psychosocial-Adolescent Version	×	
	Perinatal CARE Program Psychosocial-Parent Version		×

Caregiver drug use	Addiction Severity Index-5th Edition: Alcohol/Drug Section (modified) [83]		×
	Toxicology assays (hair and urine)		×

Caregiver psychological status	Beck Anxiety Scale (BAI) [71]		×
	Beck Depression Inventory-II (BDI) [70]		×

## References

[1] Bandstra ES, Morrow CE, Accornero VH, Mansoor E, Xue L, Anthony JC (2011). Estimated effects of in utero cocaine exposure on language development through early adolescence. *Neurotoxicology and Teratology*.

[2] Bandstra ES, Morrow CE, Anthony JC, Accornero VH, Fried PA (2001). Longitudinal investigation of task persistence and sustained attention in children with prenatal cocaine exposure. *Neurotoxicology and Teratology*.

[3] Lewis BA, Singer LT, Short EJ (2004). Four-year language outcomes of children exposed to cocaine in utero. *Neurotoxicology and Teratology*.

[4] Morrow CE, Bandstra ES, Anthony JC, Ofir AY, Xue L, Reyes ML (2001). Influence of prenatal cocaine exposure on full-term infant neurobehavioral functioning. *Neurotoxicology and Teratology*.

[5] Morrow CE, Culbertson JL, Accornero VH, Xue L, Anthony JC, Bandstra ES (2006). Learning disabilities and intellectual functioning in school-aged children with prenatal cocaine exposure. *Developmental Neuropsychology*.

[6] Messiah SE, Miller TL, Lipshultz SE, Bandstra ES (2011). Potential latent effects of prenatal cocaine exposure on growth and the risk of cardiovascular and metabolic disease in childhood. *Progress in Pediatric Cardiology*.

[7] Mone SM, Gillman MW, Miller TL, Herman EH, Lipshultz SE (2004). Effects of environmental exposures on the cardiovascular system: prenatal period through adolescence. *Pediatrics*.

[8] Richardson GA, Goldschmidt L, Larkby C (2007). Effects of prenatal cocaine exposure on growth: a longitudinal analysis. *Pediatrics*.

[9] Arendt RE, Short EJ, Singer LT (2004). Children prenatally exposed to cocaine: development outcomes and environmental risks at seven years of age. *Journal of Developmental and Behavioral Pediatrics*.

[10] Day NL, Leech SL, Richardson GA, Cornelius MD, Robles N, Larkby C (2002). Prenatal alcohol exposure predicts continued deficits in offspring size at 14 years of age. *Alcoholism*.

[11] Geva D, Goldschmidt L, Stoffer D, Day NL (1993). A longitudinal analysis of the effect of prenatal alcohol exposure on growth. *Alcoholism*.

[12] Frassica JJ, Orav EJ, Walsh EP, Lipshultz SE (1994). Arrhythmias in children prenatally exposed to cocaine. *Archives of Pediatrics and Adolescent Medicine*.

[13] Lipshultz SE, Frassica JJ, Orav EJ (1991). Cardiovascular abnormalities in infants prenatally exposed to cocaine. *Journal of Pediatrics*.

[14] Shankaran S, Lester BM, Das A (2007). Impact of maternal substance use during pregnancy on childhood outcome. *Seminars in Fetal and Neonatal Medicine*.

[15] Heron M, Hoyert DL, Murphy SL, Xu J, Kochanek KD, Tejada-Vera B (2009). Deaths: final data for 2006. *National Vital Statistics Reports*.

[16] Substance Abuse and Mental Health Services Administration (SAMHSA) *Results from the 2010 National Survey on Drug Use and Health: National Findings H-41*.

[17] McCarthy D (1972). *Manual for the McCarthy Scales of Children's Abilities*.

[18] Hollenbeck GP, Kaufman AS (1973). Factor analysis of the Wechsler preschool and primary scale of intelligence (WPPSI). *Journal of Clinical Psychology*.

[19] Folio MR, Fewell RR (1983). *Developmental Motor Scales and Activity Cards*.

[20] Beery KE (1989). *The VMI Developmental Test of Visual-Motor Integration: Administration, Scoring, and Teaching Manual*.

[21] Wechsler D (1991). *Wechsler Intelligence Scale for Children Edition*.

[22] Wechsler D (1993). *Wechsler Individual Achievement Test (WIAT)*.

[23] Wechsler D (2002). *Wechsler Individual Achievement Test (WIAT-II)*.

[24] Glutting J, Oakland : T (1992). *Guide to the Assessment of Test Session Behavior for the WISC-III and the WIAT (GATSB)*.

[25] Wiig EH, Secord W, Semel E (1992). *Clinical Evaluation of Language Fundamentals-Preschool: Examiner's Manual*.

[26] Dunn LM, Dunn LM (1981). *Peabody Picture Vocabulary Test-Revised (PPVT-R)*.

[27] Woodcock R, Johnson M (1989). *Woodcock-Johnson Psycho-Educational Battery*.

[28] Semel E, Wiig E, Secord W (1995). *Clinical Evaluation of Language Fundamentals*.

[29] Wagner RK, Torgesen JK, Rashotte CA (1999). *Comprehensive Test of Phonological Processing (CTOPP)*.

[30] Keith R (1994). *SCAN-A: A Test for Auditory Processing Disorders in Adolescents and Adults*.

[31] Korkman M, Kirk U, Kemp S (1997). *NEPSY: A Developmental Neuropsychological Assessment Manual*.

[32] Heaton R (1999). *Wisconsin Card Sorting Test: Computer Version 3*.

[33] Golden C, Freshwater S (2002). *Stroop Color and Word Test*.

[34] Reitan R, Wolfson D (1992). *Neuropsychological Evaluation of Older Children*.

[35] Bernstein JH, Waber D (1996). *Development Scoring System for the Rey-Osterrieth Complex Figure (DSS-ROCF) (the Catalog For Neuropsychological Assessment & Intervention Resources Ed.)*.

[36] Sheslow D, Adams W (1990). *Wide Range Assessment of Memory and Learning (WRAML)*.

[37] Spreen O, Strauss E (1998). *A Compendium of Neuropsychological Tests: Administration, Norms, and Commentary*.

[38] Psychological Assessment Resources Staff (1992). *Finger Tapper User's Guide*.

[39] Trites RL (1977). *Neuropsychological Test Manual*.

[40] Henderson B, Moore SG (1979). Measuring exploratory behavior in young children: a factor-analytic study. *Developmental Psychology*.

[41] Morgan GA, Busch-Rossnagel NA, Maslin-Cole CA, Harmon RJ (1992). *Individualized Assessment of Mastery Motivation: Manual for 15–36 Month Old Children*.

[42] Greenberg L, Leark R, Dupuy T, Corman C, Kindschi C, Cenedela M (1996). *Test of Variables of Attention (T.O.V.A. and T.O.V.A.A.)*.

[43] Mischel W (1974). Processes in delay of gratification. *Advances in Experimental Social Psychology*.

[44] Conners CK (1995). *Conners' Continuous Performance Test (CPT)*.

[45] Sandford JA, Turner A (1994). *Integrated Visual and Auditory Continuous Performance Test*.

[46] Achenbach TM, Rescorla L (2001). *Manual for the ASEBA School-Age Forms & Profiles*.

[47] Kovacs M (1992). *Children's Depression Inventory (CDI)*.

[48] Reynolds CR, Richmond BO (1985). *Revised Children's Manifest Anxiety Scale*.

[49] Lucas CP, Zhang H, Fisher PW (2001). The disc predictive scales (DPS): efficiently screening for diagnoses. *Journal of the American Academy of Child and Adolescent Psychiatry*.

[50] Achenbach TM (1992). *Manual for the Child Behavior Checklist/2-3 and 1992 Profile*.

[51] Conners CK (1990). *Conners' Abbreviated Symptom Questionnaire (Parent/Teacher Version) Manual*.

[52] Achenbach TM (1991). *Manual for the Child Behavior Checklist/4-18 & 1991 Profile*.

[53] Shaffer D, Fisher P, Dulcan MK (1996). The NIMH Diagnostic Interview Schedule for Children Version 2.3 (DISC- 2.3): description, acceptability, prevalence rates, and performance in the MECA study. *Journal of the American Academy of Child and Adolescent Psychiatry*.

[54] Bruininks RH, Woodcock RW, Weatherman RF, Hill BK (1996). *Scales of Independent Behavior-Revised (SIB-R)*.

[55] Reich W, Welner Z, Herjanic B (1992). *Diagnostic Interview For Children and Adolescents—IV (DICA-IV)*.

[56] Harrison PL, Oakland T (2000). *Adaptive Behavior Assessment System*.

[57] Gioia G, Isquith P, Guy S, Kenworthy L (2000). *Behavior Rating Inventory of Executive Functioning*.

[58] Gresham FM, Elliott S (1990). *SSRS: Social Skills Rating System Manual*.

[59] Egeland B, Weinfield N, Heister M

[60] Caldwell BM, Bradley RH (1984). *Manual for the Home Observation for Measurement of the Environment (H.O.M.E.) (Revised Edition)*.

[61] Richters JE, Martinez P (1990). *Things I Have Seen and Heard: An Interview for Young Children About Exposure to Violence*.

[62] (1995). *Developmental Research and Programs: Communities That Care Youth Survey*.

[63] (1990). *Department of Education and the Prevention Center, and Department of Mental Hygiene: The Baltimore, “What's Happening” Interview*.

[64] Zuckerman M, Kuhlman DM, Joireman J, Teta P, Kraft M (1993). A comparison of three structural models for personality: the big three, the big five. *Journal of Personality and Social Psychology*.

[65] Buske-Kirschbaum A, Jobst S, Wustmans A, Kirschbaum C, Rauh W, Hellhammer D (1997). Attenuated free cortisol response to psychosocial stress in children with atopic dermatitis. *Psychosomatic Medicine*.

[66] Masten A, Neemann J, Andenas S (1994). Life events and adjustment in adolescents: the significance of event independence, desirability, and chronicity. *Journal of Research on Adolescence*.

[67] Miller DB, Webster SE, MacIntosh R (2002). What’s there and what’s not: measuring daily hassles in urban African American adolescents. *Research on Social Work Practice*.

[68] Patterson JM, McCubbin HI (1987). Adolescent coping style and behaviors: conceptualization and measurement. *Journal of Adolescence*.

[69] Achenbach TM, Rescorla L (2001). *Manual for the ASEBA School-Age Forms & Profiles*.

[70] Steer RA, Beck AT, Riskind JH, Brown G (1986). Differentiation of depressive disorders from generalized anxiety by the Beck Depression Inventory. *Journal of Clinical Psychology*.

[71] Beck AT, Steer RA (1990). *Beck Anxiety Inventory Manual*.

[72] Harter S (1988). *Manual for the Self-Perception Profile for Adolescents (SPPA)*.

[73] Lejuez CW, Richards JB, Read JP (2002). Evaluation of a behavioral measure of risk taking: the balloon analogue risk task (BART). *Journal of Experimental Psychology*.

[74] Rios-Bedoya CF, Storr CL, Anthony JC Risk taking behavior in children and its association with cocaine use later in life.

[75] Barkley RA, Edwards G, Laneri M, Fletcher K, Metevia L (2001). Executive functioning, temporal discounting, and sense of time in adolescents with attention deficit hyperactivity disorder (ADHD) and oppositional defiant disorder (ODD). *Journal of Abnormal Child Psychology*.

[76] Green L, Fry AF, Myerson J (1994). Discounting of delayed rewards—a life-span comparison. *Psychological Science*.

[77] Green L, Myerson J, Lichtman D, Rosen S, Fry A (1996). Temporal discounting in choice between delayed rewards: the role of age and income. *Psychology and Aging*.

[78] Eysenck SB, Eysenck HJ (1978). Impulsiveness and venturesomeness: their position in a dimensional system of personality description. *Psychological Reports*.

[79] Ernst M, Dickstein DP, Munson S (2004). Reward-related processes in pediatric bipolar disorder: a pilot study. *Journal of Affective Disorders*.

[80] Hansen WB, Paskett ED, Carter LJ (1999). The adolescent sexual activity index (ASAI): a standardized strategy for measuring interpersonal heterosexual behaviors among youth. *Health Education Research*.

[81] Petersen AC, Crockett L, Richards M, Boxer A (1988). A self-report measure of pubertal status: reliability, validity, and initial norms. *Journal of Youth and Adolescence*.

[82] Marshall WA, Tanner JM (1969). Variations in pattern of pubertal changes in girls. *Archives of Disease in Childhood*.

[83] McLellan A, Parikh G, Bragg A, Cacciola J, Fureman B, Incmikoski R (1990). *Addiction Severity Index—administration Manual*.

[84] Bernstein DP, Fink L (1998). *Childhood Trauma Questionnaire: A Retrospective Self-Report Manual*.

[85] Moos R, Moos B (1994). *Family Environment Scale Manual (FES)*.

[86] Richters JE, Saltzman W (1990). *Survey of Exposure of Community Violence Parent Report Version*.

[87] Achenbach TM, Rescorla L (2003). *Manual For the ASEBA Adult Forms & Profiles*.

[88] Zimet GD, Powell SS, Farley GK, Werkman S, Berkoff KA (1990). Psychometric characteristics of the Multidimensional Scale of Perceived Social Support. *Journal of Personality Assessment*.

[89] Zimet GD, Dahlem NW, Zimet SG, Farley GK (1988). The multidimensional scale of perceived social support. *Journal of Personality Assessment*.

[90] Cambridge Cognition Ltd (2006). *CANTAB*.

[91] Schneiderman JF, Koren G (1990). Nonmedical drug and chemical use in pregnancy. *Maternal-Fetal Toxicology: A Clinican's Guide*.

[92] Mule SJ, Casella GA (1988). Confirmation and quantitation of cocaine, benzoylecgonine, ecgonine methyl ester in human urine by GC/MS. *Journal of Analytical Toxicology*.

[93] Brazelton TB (1984). *Neonatal Behavioral Assessment Scale*.

[94] Bayley N (1969). *Bayley Scales of Infant Development: Birth to Two Years*.

[95] Accornero VH, Morrow CE, Bandstra ES, Johnson AL, Anthony JC (2002). Behavioral outcome of preschoolers exposed prenatally to cocaine: role of maternal behavioral health. *Journal of Pediatric Psychology*.

[96] Johnson AL, Morrow CE, Accornero VH, Xue L, Anthony JC, Bandstra ES (2002). Maternal cocaine use: estimated effects on mother-child play interactions in the preschool period. *Journal of Developmental and Behavioral Pediatrics*.

[97] Bandstra ES, Morrow CE, Anthony JC, Accornero VH, Fried PA (2001). Longitudinal investigation of task persistence and sustained attention in children with prenatal cocaine exposure. *Neurotoxicology and Teratology*.

[98] Bandstra ES, Morrow CE, Mansoor E, Accornero VH (2010). Prenatal drug exposure: infant and toddler outcomes. *Journal of Addictive Diseases*.

[99] (2002). *Centers for Disease Control and Prevention (CDC). National Center for Health Statistics (NCHS). National Health and Nutrition Examination Anthropometric Procedural Manual*.

[100] Public Health Service, Centers for Disease Control and Prevention (1976). *A Proposed Method for Determining Glucose Using Hexokinase and Glucose-6-Phosphate Dehydrogenase*.

[101] (2002). American Diabetes Association: clinical practice recommendations. *Diabetes Care*.

[102] Hickman TB, Briefel RR, Carroll MD (1998). Distributions and trends of serum lipid levels among United States children and adolescents ages 4-19 years: data from the Third National Health and Nutrition Examination Survey. *Preventive Medicine*.

[103] Pearson TA, Mensah GA, Alexander RW (2003). Markers of inflammation and cardiovascular disease: application to clinical and public health practice: a statement for healthcare professionals from the centers for disease control and prevention and the American Heart Association. *Circulation*.

[104] Fernández JR, Redden DT, Pietrobelli A, Allison DB (2004). Waist circumference percentiles in nationally representative samples of African-American, European-American, and Mexican-American children and adolescents. *Journal of Pediatrics*.

[105] National High Blood Pressure Education Program Working Group on High Blood Pressure in Children and Adolescents (2004). The fourth report on the diagnosis, evaluation, and treatment of high blood pressure in children and adolescents. *Pediatrics*.

[106] Eaton DK, Kann L, Kinchen S (2006). Youth risk behavior surveillance—United States, 2005. *MMWR*.

[107] Messiah SE, Miller TL, Lipshultz SE, Bandstra ES (2011). Potential latent effects of prenatal cocaine exposure on growth and the risk of cardiovascular and metabolic disease in childhood. *Progress in Pediatric Cardiology*.

[108] Messiah SE, Arheart KL, Lipshultz SE, Miller TL (2008). Body mass index, waist circumference, and cardiovascular risk factors in adolescents. *Journal of Pediatrics*.

